# A nonlinear viscoelastic constitutive model with damage and experimental validation for composite solid propellant

**DOI:** 10.1038/s41598-023-29214-7

**Published:** 2023-02-04

**Authors:** Hui Li, Jin-sheng Xu, Xiong Chen, Jun-fa Zhang, Juan Li

**Affiliations:** 1 School of Mechanical Engineering, Nanjing University of Science and Technology, Nanjing, 210094 People’s Republic of China; 2 Beijing Institute of Space Long March Vehicle, Beijing, 100070 People’s Republic of China; 3 Xi’an Changfeng Research Institute of Mechanism and Electricity, Xi’an, 710065 People’s Republic of China

**Keywords:** Aerospace engineering, Mechanical properties

## Abstract

The development of a nonlinear viscoelastic constitutive model of composite solid propellant (CSP) coupled with effects of strain rate and confining pressure is essential to assess the reliability of solid propellant grains during ignition operation process. In the present work, a nonlinear viscoelastic constitutive model with novel energy-based damage initiation criterion and evolution model was firstly proposed to describe the coupled effects of confining pressure and strain rate on mechanical responses of CSP. In the developed damage initiation criterion and evolution model, the linear viscoelastic strain energy density was introduced as the damage driving force, and the coupled effects of strain rate, damage history and confining pressure on damage growth were taken into account. Then, uniaxial tensile tests from low strain rates to medium strain rates and various confining pressures, and stress relaxation tests were conducted using a self-made active confining pressure device. Finally, the identification procedures of model parameters and validation results of the constitutive model were presented. Moreover, the master curve of damage initiation parameter was constructed through the time-pressure superposition principle (TPSP). The results show that the developed nonlinear constitutive model is capable of predicting the stress–strain responses of CSP under different strain rates and confining pressures.

## Introduction

Since the advantage of high energy density and easy storage, composite solid propellant (CSP) is widely used to be the propulsive source of solid rocket motors (SRMs). In general, CSP is composed by a viscoelastic polymer binder system embedded with a large number of solid particles (e.g., ammonium perchlorate, AP, aluminum, Al). During the service life of CSP grains, they will be subjected to various loads, such as the temperature load from change of environmental conditions, vibration load from transportation, and pressure load from ignition pressurization process. Under these loads, the microstructure of CSP changes, including dewetting along interfaces between filler particles and binder, and nucleation and growth of micro-voids^1,2^. As a result, CSP usually exhibits nonlinear and complex mechanical behaviors in macroscopic level. The performance of a SRM is significantly influenced by the structural integrity of CSP grains. Comparing to other loads, CSP grains are most prone to fail during ignition pressurization process. Under ignition pressurization load, CSP grains are in a tri-axial compression stress state (confining pressure state) by the gas, and their mechanical responses are significantly different from those at room condition. As a typical viscoelastic material, mechanical responses of CSP are strongly dependent on strain rate and environmental pressure condition. It reveals that these constitutive models validated under room pressure cannot accurately predict mechanical responses of propellant grains during ignition process^3–5^. Therefore, it is of great importance to develop a nonlinear constitutive model incorporated the coupled effects of strain rate and confining pressure, and conduct corresponding experimental validation to reveal these complex mechanical performances and further assess the reliability of CSP grains during ignition operation process.

Over the past decades, a few researchers have developed a few constitutive models of solid propellant considering the effect of confining pressure. One of the earliest available reports to characterize the effect of pressure on stress–strain behaviors has been done by Farris^6^. He derived the stress–strain function for highly filled elastomers using a simple thermodynamic model. Swanson et al.^7^ indicated the effect of pressure on the strain-softening function by fitting experimental data. Based on a work potential theory and a micromechanical model^8^, Schapery^9,10^ developed a constitutive model to characterize the nonlinear elastic deformation behaviors of solid propellant under axial tension and confining pressure. Later, Park and Schapery^11,12^ extended the above model to a thermo-viscoelastic model using the so-called pseudo strain theory, time–temperature superposition principle (TTSP) and rate-type evolution equation of two internal damage variables, which can model the effects of axial strain rate, temperature and confining pressure on Hydroxy-Terminated Polybutadiene (HTPB) propellant. Furthermore, Ha and Schapery^13^, and Hinterhoelzl and Schapery^14^ have successively extended the model theory of Park and Schapery^11,12^ to three dimensions and implemented it in Abaqus software. Ravichandran and Liu^15^ proposed a simple rate-independent phenomenological constitutive model with two damage functions related to the degradation of the bulk and shear modulus. The effect of confining pressure on uniaxial response was investigated and the stress–strain responses under various pressures (0–2 MPa) were presented. Özüpek et al.^16,17^ developed three initial isotropic constitutive models, and introduced an exponential function with a pressure term into the function of growth rate of void volume fraction caused by detwetting damage to model suppression effect of pressure on damage growth of Polybutadiene-Acrylonitrile (PBAN) propellant. The predicted results do not agree with the experimental data well under high strain rate due to assumption that damage is rate-independent. Canga et al.^18^ modified the model to allow an efficient numerical implementation and presented the comparisons between finite element analysis results and test data.

In recent years, following Simo’s^19^ finite strain framework, as the total strain energy is decomposed into deviatoric and dilational parts, Tunç and Özüpek^20,21^ constructed and modified a three-dimensional damaging finite strain viscoelastic model and implemented it in Abaqus software as a user material subroutine. The exponential function proposed by Özüpek et al.^16,17^ was also adopted to represent the effect of confining pressure on solid propellant. Supposing that damage evolution obeyed the Weibull probability distribution function and damage parameters were pressure-dependent, Li et al.^22,23^ proposed two kinds of nonlinear viscoelastic models with damage to model the effect of pressure on tension and compression behaviors of Nitrate Ester Plasticized Polyether (NEPE) propellant. He pointed out that the confining pressure can delay or suppress the damage initiation and growth. Kantor et al.^24^ developed a three-dimensional hyper viscoelastic equation and proposed a new strain rate, damage rate and stress state sensitive dewetting (damage) criterion. The model was implemented into MSC. Marc by means of Fortran user subroutine, and calibrated and validated through the experimental data provided by Park and Schapery^11,12^.

**Figure 1:**
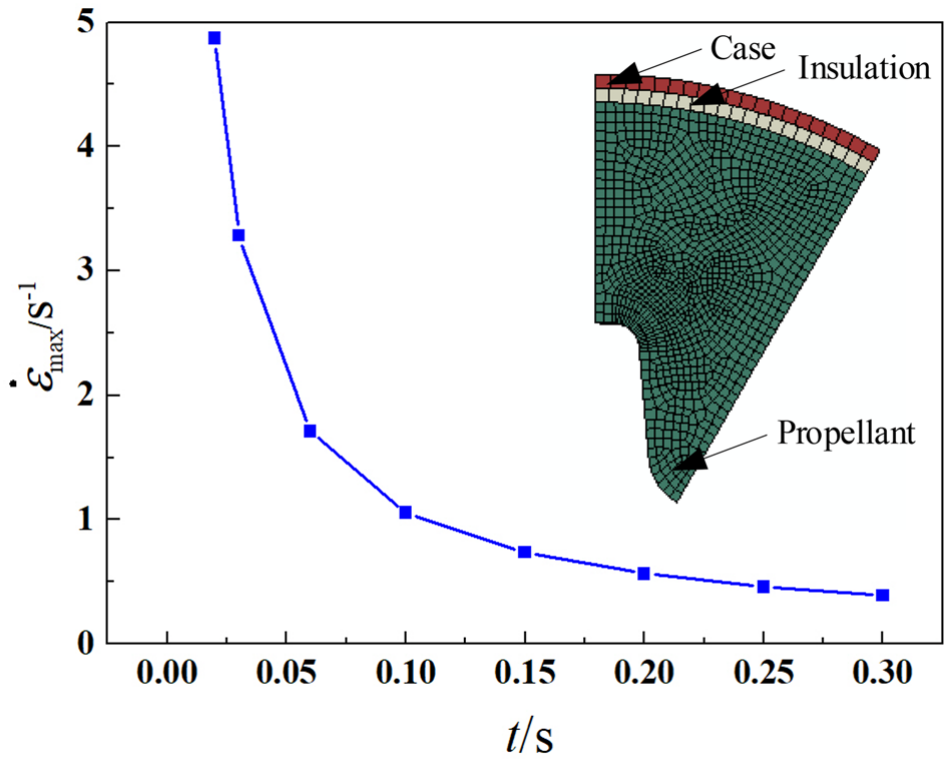
Maximum circumferential tensile strain rate of a typical star-hole grain when pressurized to 15 MPa at different times (0.03–0.3 s) calculated by Abaqus software.

In summary, although these developed constitutive models accessed from the above literatures have made a great progress in describing the nonlinear behaviors of solid propellant under the coupled effects of confining pressure and strain rate, there is still a great lack of research. On the one hand, the model parameters calibration procedures are complex. A few models need strain-dilatation data under various confining pressures to identify the model parameters, which is difficult to be obtained^6,11,12,20,21,24^. Thence, these models would face a great difficulty in engineering application. On the other hand, the experimental data used to verify these models in literatures involve confining pressure less than 6 MPa and the strain rate lower than 0.5 s^−1^. However, for a realistic SRM, the maximum pressure around CSP grains is about 8–10 MPa and the maximum strain rate is larger than 0.5 s^−1^ during ignition operation process (see Fig. 1). Due to the lack of related experimental data, these model validation results cannot demonstrate the predictive capability under a realistic extreme condition of propellant grains^6,11,12,20–24^. Therefore, it is meaningful to present the experimental data of modern CSP under the above mentioned extremer conditions, which will be one of the work in this paper.

The main objective of this paper is to develop a nonlinear viscoelastic constitutive model with damage to describe the coupled effects of strain rate and confining pressure on mechanical responses for CSP. Firstly, based on the framework of irreversible thermodynamic theory, a nonlinear viscoelastic constitutive model with damage was proposed. Meanwhile, the damage initiation criterion and evolution model were developed. Furthermore, through the self-made active confining pressure experimental device, the mechanical responses of CSP under different strain rates and confining pressures were obtained. Finally, the model parameters were calibrated, and the comparisons of model predictions and experimental data were presented.

## Constitutive model

### Basic theory of thermodynamic

For a dissipative viscoelastic material system, its state law of thermodynamics can be characterized through a few external variables, e.g., temperature $T$, strain ${\varvec{\varepsilon}}$, temperature gradient ${\mathbf{\nabla }}T$, and a series of internal state variables (ISVs, such as damage variable $D$ and hardening variable $R$). Under an external load, the internal microstructure of the material changes resulting in changes with the ISVs, which is regarded as the main reason to cause the nonlinear mechanical responses of viscoelastic material. This process is generally considered as an irreversible energy dissipation process, and it should meet the first and second law of thermodynamics.

The first law of thermodynamic or the law of conservation of energy can be described as^25^

1 $$\rho \dot{e} - {\varvec{\sigma}}:\dot{\user2{\varepsilon }} + {\mathbf{\nabla }} \cdot {\varvec{q}} - \rho \gamma = 0$$

where $\rho$, $e$, ${\varvec{\sigma}}$, $\dot{\user2{\varepsilon }}$, ${\mathbf{\nabla }}$, ${\varvec{q}}$ and $\gamma$ are mass density, the specific internal energy, the stress tensor, the differential of strain tensor ${\varvec{\varepsilon}}$ with respect to time $t$, the gradient operator, the heat flux density vector and the specific heat supply rate, respectively. In addition, $e = \varphi + Ts$, where $\varphi$ is the Helmholtz free energy and $s$ is specific entropy.

The second law of thermodynamic or the Clausius–Duhem inequality can be presented by

2 $$\rho T\dot{s} + {\mathbf{\nabla }} \cdot {\varvec{q}} - \frac{{\varvec{q}}}{T} \cdot {\mathbf{\nabla }}T - \rho \gamma \ge 0$$

Substituting Eq. (1) into Eq. (2) to eliminate the specific heat supply rate, then

3 $${\varvec{\sigma}}:\dot{\user2{\varepsilon }} - \rho \dot{\varphi } - \frac{{\varvec{q}}}{T} \cdot {\mathbf{\nabla }}T \ge 0$$

**Figure 2:**
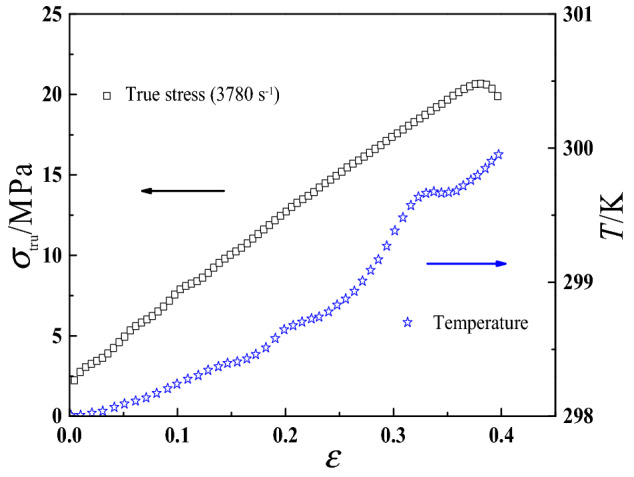
Stress–strain-temperature curves for HTPB propellant^26^.

Figure 2 shows the variation of internal temperature of HTPB propellant under impact strain rate of 3780 s^−1^, which is less than 3 K^26^. Thence, it can be assumed that, under quasi-static load (≤ 1 s^−1^) and medium strain rate load (1–100 s^−1^), the self-heating effect caused by the deformation is very small for propellant material, and the influence of internal temperature change can be ignored. Isothermal conditions are assumed in this work, so the above formula Eq. (3) can be simplified as

4 $${\varvec{\sigma}}:\dot{\user2{\varepsilon }} - \rho \dot{\varphi } \ge 0$$

In this work, it is assumed that the soft CSP has only viscoelastic deformation under external load, and it is accompanied by the initiation and growth of damage. Introducing the internal isotropic scalar damage variable $D$, the Helmholtz free energy $\varphi$ can be assumed as a coupled function of the viscoelastic deformation ${\varvec{\varepsilon}}^{{{\text{ve}}}}$ and internal damage variable $D$.

5 $$\varphi { = }\varphi^{{{\text{ve}}}} ({\varvec{\varepsilon}}^{{{\text{ve}}}} ,D)$$

The total differential of the Helmholtz free energy $\varphi$ with respect to $t$ is

6 $$\dot{\varphi }{ = }\frac{{\partial \varphi^{{{\text{ve}}}} }}{{\partial {\varvec{\varepsilon}}^{{{\text{ve}}}} }}\dot{\user2{\varepsilon }}^{{{\text{ve}}}} + \frac{{\partial \varphi^{{{\text{ve}}}} }}{\partial D}\dot{D}$$

Inserting the Eq. (6) into Eq. (4), then

7 $$\left( {{\varvec{\sigma}} - \rho \frac{{\partial \varphi^{{{\text{ve}}}} }}{{\partial {\varvec{\varepsilon}}^{{{\text{ve}}}} }}} \right):\dot{\user2{\varepsilon }}^{{{\text{ve}}}} - \rho \frac{{\partial \varphi^{{{\text{ve}}}} }}{\partial D}\dot{D} \ge 0$$

Since the inequality Eq. (7) needs to be satisfied for an arbitrary value of $\dot{\user2{\varepsilon }}^{{{\text{ve}}}}$, the coefficient should be zero, then,

8 $${\varvec{\sigma}} = \rho \frac{{\partial \varphi^{{{\text{ve}}}} }}{{\partial {\varvec{\varepsilon}}^{{{\text{ve}}}} }}$$

Meanwhile, the damage dissipation rate or the energy density release rate can be defined as

9 $$Y = - \rho \frac{{\partial \varphi^{{\text{ve }}} }}{\partial D}$$

where the $Y$ and $D$ are a pair of conjugate thermodynamic forces. Assuming that the dissipation potential $\phi$ only has the damage part, we can get

10 $$\phi = \phi^{{\text{d}}} (Y,D) = Y\dot{D} \ge 0$$

where $\phi^{{\text{d}}}$ is damage dissipation potential. The rate of the damage variable is given as

11 $$\dot{D} = \frac{{\partial \phi^{{\text{d}}} }}{\partial Y}$$

### Concept of effective stress

The concept of effective stress is introduced by the Kachanov^27^ and Rabotnov^28^ to solve the life prediction of metal, and further developed to 3D states by the Lemaitre^29^, as well as other researchers. For a material specimen under external load condition, when the occurrence of the damage with the increasing of load, the cross-sectional area is $A$ and the applied stress tensor is called as nominal stress tensor ${\varvec{\sigma}}$. Meantime, considering a fictitious configuration of the material specimen without damage, which is obtained from the damaged configuration by removing all the damage, its effective cross-sectional area is $A_{{{\text{eff}}}}$ and the stress in the fictitious configuration is named as the effective stress tensor $\tilde{\user2{\sigma }}$. The isotropic scalar damage variable can be defined as follows

12 $$D = \frac{{A - A_{{{\text{eff}}}} }}{A};{\kern 1pt} {\kern 1pt} {\kern 1pt} {\kern 1pt} {\kern 1pt} {\kern 1pt} {\kern 1pt} {\kern 1pt} {\kern 1pt} {\kern 1pt} 0 \le D \le 1$$

Let a same body force act on the damaged configuration and fictitious configuration, that is, ${\varvec{\sigma}}{\rm A}\user2{ = \tilde{\sigma }}{\rm A}_{{{\text{eff}}}}$. Then relation between the effective stress $\tilde{\user2{\sigma }}$ and nominal stress ${\varvec{\sigma}}$ can be obtained as^30^

13 $$\tilde{\user2{\sigma }} = \frac{{\varvec{\sigma}}}{1 - D}$$

### Thermodynamic derivation of viscoelastic model with damage

Previous studies have proved that there is no damage inside the solid propellant under a small loading, the propellant material obeys the linear viscoelasticity theory^22,31,32^. As the load continues, damage such as microcracks and interface debonding appear, which leads to a nonlinear mechanical response. Therefore, the deformation problem of the solid propellant can be regarded as a viscoelastic media coupled with damage.

Considering an elastic media coupled with damage, the Helmholtz free energy $\varphi^{{\text{e}}}$ can be written as^33^:

14 $$\rho \varphi^{{\text{e}}} \left( t \right) = \frac{1}{2}\left( {1 - D} \right){\varvec{\varepsilon}}^{{\text{e}}} :{\varvec{C}}^{{\text{e}}} :{\varvec{\varepsilon}}^{{\text{e}}}$$

where $D$ is the isotropic scalar damage variable, ${\varvec{\varepsilon}}^{{\text{e}}}$ is the elastic strain tensor, and ${\varvec{C}}^{{\text{e}}}$ is the fourth order undamaged elasticity modulus tensor.

Similarly to the expression of elastic media coupled with damage, the Helmholtz free energy of viscoelastic media coupled with damage is defined as^34^:

15 $$\rho \varphi^{{{\text{ve}}}} \left( t \right) = \frac{1}{2}\left( {1 - D\left( t \right)} \right)\int_{ - \infty }^{t} {\int_{ - \infty }^{t} {\frac{{\partial {\varvec{\varepsilon}}^{{{\text{ve}}}} \left( \tau \right)}}{\partial \tau }} } :{\varvec{C}}^{{{\text{ve}}}} \left( {2t - \tau - \eta } \right):\frac{{\partial {\varvec{\varepsilon}}^{{{\text{ve}}}} \left( \eta \right)}}{\partial \eta }{\text{d}}\tau {\text{d}}\eta$$

where ${\varvec{C}}^{{{\text{ve}}}} (t)$ is the fourth order undamaged relaxation modulus tensor. Using the particular form ${\varvec{C}}^{{{\text{ve}}}} (\tau ,\eta ) = {\varvec{C}}^{{{\text{ve}}}} (\tau + \eta )$, and combing the inequality Eq. (7), the damaged stress ${\varvec{\sigma}}\left( t \right)$ can be obtained through the total differential of with $\rho \varphi^{{{\text{ve}}}} (t)$ respect to time $t$:

16 $${\varvec{\sigma}}\left( t \right) = \rho \frac{{\partial \varphi^{{{\text{ve}}}} }}{{\partial {\varvec{\varepsilon}}^{{{\text{ve}}}} }} = \left( {1 - D\left( t \right)} \right)\int_{ - \infty }^{t} {{\varvec{C}}^{{{\text{ve}}}} \left( {t - \tau } \right)} :\frac{{\partial {\varvec{\varepsilon}}^{{{\text{ve}}}} \left( \tau \right)}}{\partial \tau }{\text{d}}\tau$$

According to the Eqs. (13) and (16), the effective stress $\tilde{\user2{\sigma }}\left( t \right)$ is given as

17 $$\tilde{\user2{\sigma }}\left( t \right) = \int_{ - \infty }^{t} {{\varvec{C}}^{ve} \left( {t - \tau } \right)} :\frac{{\partial {\varvec{\varepsilon}}^{{{\text{ve}}}} \left( \tau \right)}}{\partial \tau }{\text{d}}\tau$$

The Eq. (17) shows that the effective stress is same with linear viscoelastic stress. Then, based on the Eq. (9) and Eq. (15), the damage dissipation rate or damage thermodynamic force $Y(t)$ can be obtained

18 $$Y\left( t \right) = - \rho \frac{{\partial \varphi^{{{\text{ve}}}} }}{\partial D} = \frac{1}{2}\int_{ - \infty }^{t} {\int_{ - \infty }^{t} {\frac{{\partial {\varvec{\varepsilon}}^{{{\text{ve}}}} \left( \tau \right)}}{\partial \tau }} } :{\varvec{C}}^{{{\text{ve}}}} \left( {t - \tau ,t - \eta } \right):\frac{{\partial {\varvec{\varepsilon}}^{{{\text{ve}}}} \left( \eta \right)}}{\partial \eta }{\text{d}}\tau {\text{d}}\eta$$

It shows that the damage dissipation rate can be interpreted as the linear viscoelastic strain energy density.

### Novel energy-based damage model

Different damage models have been proposed to predict the damage initiation and growth of viscoelastic materials under various loads. Kachanov^27^ pioneered that the creep damage is a function of stress and damage history. Schapery^35^ found that the local crack growth speed obeys a power law in local stress intensity or J-integral. Guided by the local crack growth equation, he proposed a rate-type evolution law for ISVs in viscoelastic media. Based on the cumulative damage theory, the damage was presented in terms of the integral of stress history with respect to time by Duncan and Margetson^36^. Besides, in order to make the established damage model meet the basic principles of thermodynamics, many researchers often define different forms of damage-based dissipation potential functions to derive different internal variable evolution laws. Chen et al.^37^ defined the damage-based dissipation potential as a temperature-dependent function and described the damage evolution behaviors of asphalt materials at different temperatures. Similarly, Abu Al-Rub et al.^38^ considered the difference between tension and compression damage evolution and the influence of temperature on damage evolution, and proposed the rate of thermo-viscodamage dissipation as a function of temperature and effective strain.

Motivated and guided by the aforementioned work, the damage evolution law can be determined by defining the damage dissipation potential with confining pressure and strain rate in this work. Nevertheless, before describing damage evolution, a damage initiation criterion needs to be determined. For example, based on the Jung’s^39^ viscoelastic dewetting criterion, Yun et al.^40^ derived a simplified viscoelastic dewetting function, which supposes that when the second deviatoric stress invariant reaches a specific temperature-dependent constant, the dewetting damage of solid propellant appears. However, since the viscoelastic dewetting damage function does not consider the effect of strain rate, a few overpredictions can be observed at low strain rate. In this work, following the energy-based damage framework of Lemaitre^41^ and Onifade^42^, the damage initiation criterion for CSP is also defined via the damage initiation potential function $\varphi_{1}^{*}$ as^42^

19 $$f_{{\text{d}}} = \varphi_{1}^{*} (Y) - \varphi_{{1,{\text{c}}}}^{*} \left( {S_{0} } \right){ = }0$$

where $\varphi_{1}^{*} \left( Y \right)$ is damage initiation potential function, $\varphi_{{1,{\text{c}}}}^{*} \left( {S_{0} } \right)$ is the critical value of damage initiation potential. In Onifade’s work, the damage initiation potential function $\varphi_{1}^{*} \left( Y \right)$ was defined as the power function of the linear viscoelastic strain energy density^42^

20 $$\varphi_{1}^{*} = \frac{{S_{0} }}{{k_{1} + 1}} \cdot \left( {\frac{Y}{{S_{0} }}} \right)^{{k_{1} + 1}} \cdot \frac{1}{1 - D}$$

where $k_{1}$ is material constant, $S_{0}$ is damage initiation parameter, $Y$ is linear viscoelastic strain density and can be regarded as damage driving force. It shows that as the damage driving force $Y$ increases with external load and when the damage driving force $Y$ equals damage initiation parameter $S_{0}$ or the damage initiation potential reaches a critical value $\varphi_{{1,{\text{c}}}}^{*} \left( {S_{0} } \right)$, damage would initiate and the mechanical response would change from linear response to nonlinear response.

However, in this work, to describe the effects of strain rate and confining pressure on damage initiation, the damage initiation potential function $\varphi_{1}^{*} \left( Y \right)$ is further defined as the function of strain rate and confining pressure, and expressed as

21 $$\varphi_{1}^{*} = \frac{{S_{0} }}{{k_{1} + 1}} \cdot \left( {\frac{Y}{{S_{0} }}} \right)^{{k_{1} + 1}} \cdot g(D) \cdot \left( {\frac{{\dot{\varepsilon }}}{{\dot{\varepsilon }_{0} }}} \right) \cdot \vartheta \left( p \right)$$

where damage initiation parameter $S_{0}$ is assumed to depend on strain rate and confining pressure. To unify the dimensions, the reference strain rate $\dot{\varepsilon }_{0}$ is introduced, and without plastic deformation, $\dot{\varepsilon }{ = }\dot{\varepsilon }^{{{\text{ve}}}}$. The function $g(D)$ is used to describe the influence of damage history on the damage evolution. Note that when $D = 0$ (i.e. no damage), $g(0){ = }1$. In addition, the function $\vartheta (p)$ is used to characterize the effect of confining pressure on damage initiation and growth. According to Eq. (19) and Eq. (21), the critical value of damage initiation potential can be presented as

22 $$\varphi_{{1,{\text{c}}}}^{*} { = }\frac{{S_{0} }}{{k_{1} + 1}} \cdot \left( {\frac{{\dot{\varepsilon }}}{{\dot{\varepsilon }_{0} }}} \right) \cdot \vartheta \left( p \right)$$

It shows that comparing with traditional damage initiation criterion with critical strain^22,43^ or critical stress^40,44^ as the judgment parameter, the new damage initiation criterion (see Eqs. (19) and (21)) adopts the strain energy density as the judgement parameter, which takes into account both the stress and strain.

In general speaking, there are two methods to characterize the influence of damage history on damage growth, that is the function $\left( {1 - D} \right)^{n}$ used as the numerator or denominator, such as:

23 $$g(D) = \left\{ \begin{gathered} \frac{1}{{\left( {1 - D} \right)^{n} }} \hfill \\ \left( {1 - D} \right)^{n} \hfill \\ \end{gathered} \right.$$

where $n$ is material parameter, which characterizing the sensitivity of damage evolution to damage history. The first method indicates that as the value of damage increases, the damage growth rate increases, which will lead a brittle fracture behavior. The second method shows that as the value of damage increases, the damage growth rate decreases, which will lead a ductile fracture behavior. Since stress–strain curves of CSP usually have an obvious “plateau” stage (except for the low temperature and high strain rate condition), it exhibits ductile fracture behavior^11,22^. Therefore, the second method is adopted in this work.

**Figure 3:**
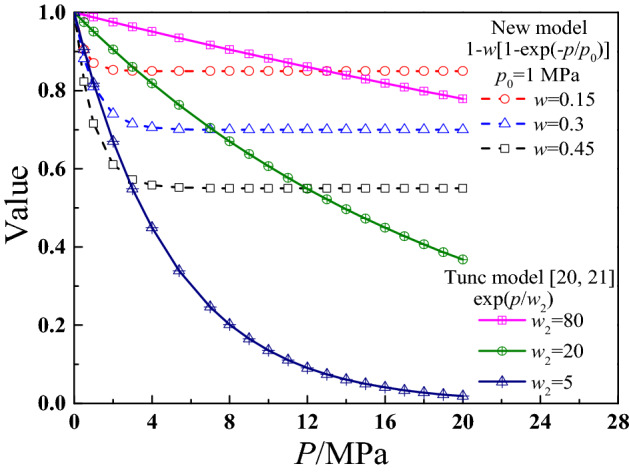
Comparisons of the two confining pressure effect functions.

Previous studies have pointed out that confining pressure can delay the appearance of dewetting and micro-cracks inside CSP, and limit the expansion in the adhesive surrounding the solid filler^22,45–47^. Özüpek^16,17^ and Tunç^20,21^ introduced the effect of confining pressure from the perspective that confining pressure will affect growth rate of voids caused by dewetting of solid filler particles. An exponential expression including the pressure term was proposed as^20,21^

24 $$\dot{c}(t) = \dot{\gamma }(t)\exp \left( {p/w_{1} } \right)$$

where $\dot{c}\left( t \right)$ is growth rate of voids, $\gamma \left( t \right)$ accounts for the influence of distortional deformation, while the exponential term $\exp \left( {p/w_{1} } \right)$ represents the confining pressure effect and $w_{1}$ is material parameter. The lower the value of this term ($\exp \left( {p/w_{1} } \right)$), the larger the effect of confining pressure on the suppression of voids growth. Due to the voids are the specific manifestation of damage, it also means that the damage suppression effect of confining pressure increases with a increasing of confining pressure. However, the exponential term also shows that if confining pressure keeps increasing, its value decreases and the suppression effect of confining pressure on growth rate of voids or damage would keep increasing, as shown in Fig. 3.

According to experimental observation, Traissac et al.^45^ indicated that there is a saturation confining pressure value, that is, when confining pressure condition exceeds the saturation pressure value, the mechanical properties of solid propellant would not change significantly as confining pressure increases. In another words, after the saturation pressure is exceeded, the pressure has no further suppression effect on damage growth rate of solid propellant, and the value of the exponential term in Eq. (24) or $\vartheta (p)$ defined in this paper should not continue to decrease obviously with increasing confining pressure. However, it can be observed from the Fig. 3 that the exponential term proposed and adopted by Özüpek^16,17^ and Tunç^20,21^ cannot describe the existence of saturation pressure, and the Refs.^20,21^ also show that the exponential term does not present the nonlinear relationship between confining pressures and the corresponding performance changes of solid propellant well. Li et al.^46^ regarded the value of saturation confining pressure is between 5 and 7 MPa, Bihari et al.^47^ pointed out that the value of saturation confining pressure is between 4 and 6 MPa and Wang et al.^48^ found it is between 0.15–4 MPa. Therefore, based on the experimental observation, another empirical exponential function to capture the effect of confining pressure and saturation confining pressure is proposed in this paper, which has the following form

25 $$\vartheta (p) = 1 - w \cdot \left( {1 - \exp \left( { - \frac{p}{{p_{0} }}} \right)} \right)$$

where $w$ is material parameter and determined by experimental data, and the parameter $p_{0}$ is introduced to make dimensionless and can be regarded as a reference parameter. In addition, Fig. 3 also shows that the value of the Eq. (25) varies with confining pressure. It can be found that the three curves maintain constant after confining pressure exceeds 5 MPa, which is close to the value of saturation pressure discussed in the literatures^46–48^. It demonstrates that the confining pressure function proposed in this work can describe the law that under low confining pressure condition, the pressure has an obvious suppression effect on damage, and when the saturation pressure is reached, the suppression effect is basically unchanged.

Using the non-associated damage evolution rule, the damage evolution criterion (damage-based dissipation potential) is presented as^42^

26 $$\phi^{{\text{d}}} = f_{{\text{D}}} = \varphi_{2}^{*} \left( Y \right) - \alpha \cdot \varphi_{{1,{\text{c}}}}^{*} \left( {S_{0} } \right) > 0$$

where $\alpha = k_{2} /k_{1}$, $k_{2}$ is material parameter. $\varphi_{2}^{*}$ is damage growth potential function, and based on Eq. (21), it is defined as

27 $$\varphi_{2}^{*} = \alpha \varphi_{1}^{*} = \frac{{k_{2} }}{{k_{1} }} \cdot \frac{{S_{0} }}{{k_{1} + 1}} \cdot \left( {\frac{Y}{{S_{0} }}} \right)^{{k_{1} + 1}} \cdot \left( {\frac{{\dot{\varepsilon }}}{{\dot{\varepsilon }_{0} }}} \right) \cdot (1 - D)^{n} \cdot \left[ {1 - w \cdot \left( {1 - \exp \left( { - \frac{p}{{p_{0} }}} \right)} \right)} \right]$$

Substituting Eq. (27) into Eq. (11), which yields:

28 $$\dot{D} = \frac{{k_{2} }}{{k_{1} }} \cdot \left( {\frac{Y}{{S_{0} }}} \right)^{{k_{1} }} \cdot \left( {\frac{{\dot{\varepsilon }}}{{\dot{\varepsilon }_{0} }}} \right) \cdot (1 - D)^{n} \cdot \left[ {1 - w \cdot \left( {1 - \exp \left( { - \frac{p}{{p_{0} }}} \right)} \right)} \right]$$

The damage evolution law can be presented as.

(I) If ${\kern 1pt} \varphi_{1}^{*} \left( Y \right) < \varphi_{1,c}^{*} \left( {S_{0} } \right)$, no damage.
(II) If $\varphi_{1}^{*} \left( Y \right){ = }\varphi_{1,c}^{*} \left( {S_{0} } \right)$, damage initiation.
(III) If $\varphi_{2}^{*} \left( Y \right) > \alpha \cdot \varphi_{1,c}^{*} \left( {S_{0} } \right)$, damage accumulation, $\dot{D} = \frac{{k_{2} }}{{k_{1} }} \cdot \left( {\frac{Y}{{S_{0} }}} \right)^{{k_{1} }} \cdot \left( {\frac{{\dot{\varepsilon }}}{{\dot{\varepsilon }_{0} }}} \right) \cdot (1 - D)^{n} \cdot \left[ {1 - w \cdot \left( {1 - \exp \left( { - \frac{p}{{p_{0} }}} \right)} \right)} \right]$.

## Materials and experiments

The nonlinear constitutive model considering confining pressure and strain rate was developed in last section. In this section, the experimental material and experimental method will be introduced to identify model parameters.

### Materials and specimens

**Figure 4:**
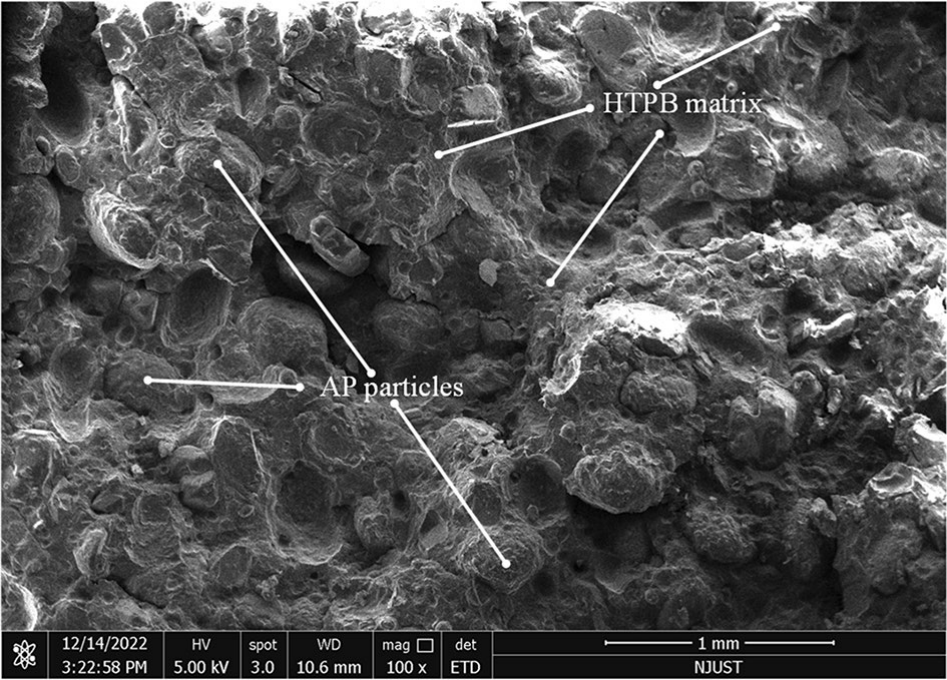
Scanning electron microscope (SEM) image of tested propellant.

The experimental material used in this investigation is a kind of typical three component Hydroxy-Terminated Polybutadiene (HTPB) propellant, which is composed of 60–70%-wt% of AP (ammonium perchlorate) particles, 10–20%-wt% of a HTPB matrix and other additives including Al (aluminum) particles and RT-01 catalyst. The scanning electron microscope (SEM) image of tested propellant is shown as Fig. 4, which reveals the diameter of AP particles are about 200 μm.

According to the standard of P. R. C. GJB 770B-2005 test method of propellant, the HTPB propellant was designed as a dumbbell-shaped, including a gauge length of 70 ± 0.5 mm, a width of 10 ± 0.5 mm, and a thickness of 10 ± 0.5 mm.

### Experimental system

**Figure 5:**
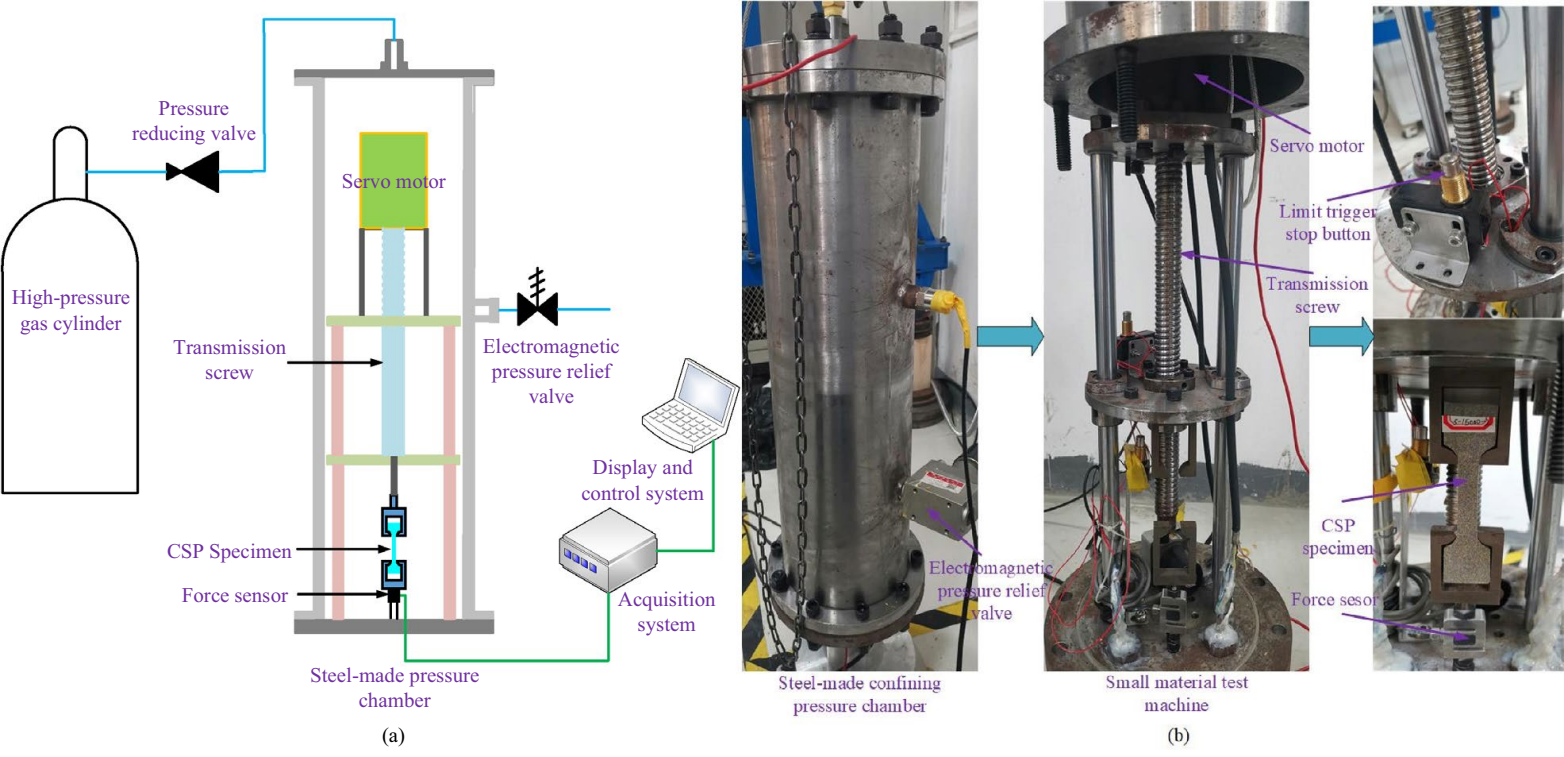
New active confining pressure experimental system. (**a**) Schematic diagram and (**b**) actual diagram.

In this work, comparing to the experimental system showed in Ref.^22^, a new self-made active confining pressure system was developed to reach higher strain rate and confining pressure condition. As shown in Fig. 5a, the experimental system includes a high-pressure gas cylinder part, a steel-made pressure chamber, a small self-made uniaxial tensile machine and a control and acquisition system. The small self-made uniaxial material test machine is driven by a servo motor, and the CSP specimen is stretched through the transmission screw. Besides, the displacement is measured by a rope-type displacement sensor, and its accuracy can reach 0.01 mm. Figure 5b shows the actual structure of experimental system. The maximum stretching speed is 15,000 mm/min and the range of force sensor is 2000 N. The servo motor drives the tested propellant specimen through the transmission screw. The steel-made pressure chamber can withstand the highest pressure of 15 MPa.

### Uniaxial tensile tests and relaxation tests

According to previous experimental results^11,17,22,47^, to accurately reflect the coupled effects of confining pressure and strain rate on mechanical properties of CSP, five groups of confining pressure conditions with relative atmospheric pressure of 0 MPa (room pressure), 0.5 ± 0.05 MPa, 2 ± 0.05 MPa, 5 ± 0.05 MPa, 8 ± 0.1 MPa applied by nitrogen gas were selected. Meantime, five groups of uniaxial tensile speeds tests of 50 mm/min, 200 mm/min, 1000 mm/min, 5000 mm/min and 15,000 mm/min (the corresponding strain rate is 1.190 × 10^–2^ s^−1^, 4.762 × 10^–2^ s^−1^, 2.381 × 10^–1^ s^−1^, 1.190 s^−1^ and 3.571 s^−1^) were carried out under each confining pressure condition to identify the model parameters and validate the constitutive model.

In order to obtain the linear viscoelastic parameters and determine the transformation point of CSP from linear response to nonlinear response, the stress relaxation tests were carried out. Due to confining pressure has no significant influence on elastic modulus of the CSP, it can be supposed that the linear viscoelastic parameters are same under various confining pressures. The CSP specimens were firstly preloaded 3 N, then stretched to a strain of 0.06 with a strain rate of 1.190 × 10^–1^ s^−1^, and the strain was kept constant for 1200 s at room condition. Meanwhile, the acquisition system record the variation of force and time during the experimental process.

Due to the mechanical properties of CSP is sensitive to temperature and the temperature is not considered in this work, the whole tests were performed at 298 ± 3 K. To guarantee the validity and repeatability of experimental results, the thickness and width of each CSP specimen should be measured before stating the test, and tension tests should be repeated at least three times under each test condition.

## Model parameters identification and validation

### Identification of model parameters

**Figure 6:**
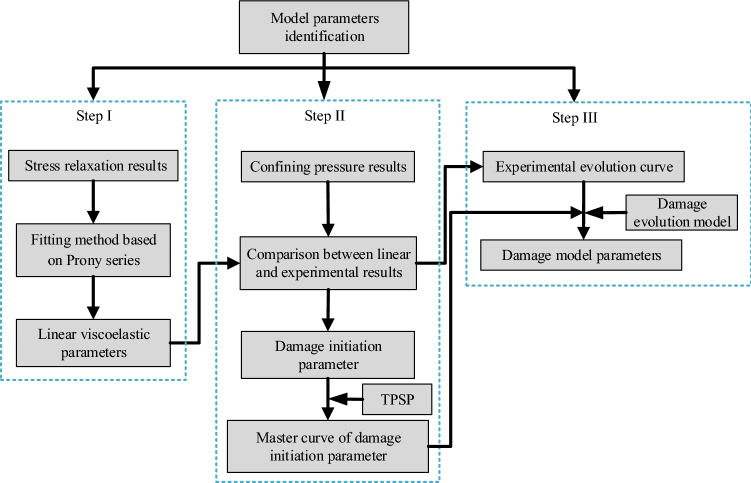
The flow chart of model parameters identification.

In the developed model, the model parameters including linear viscoelastic parameters, damage initiation parameter and damage evolution model parameters need to be identified, which can be acquired by stress relaxation results and uniaxial constant strain rate results performed in section “Uniaxial tensile tests and relaxation tests”. The whole model parameters identification process is shown in Fig. 6.

#### Identification of linear viscoelastic parameters

**Figure 7:**
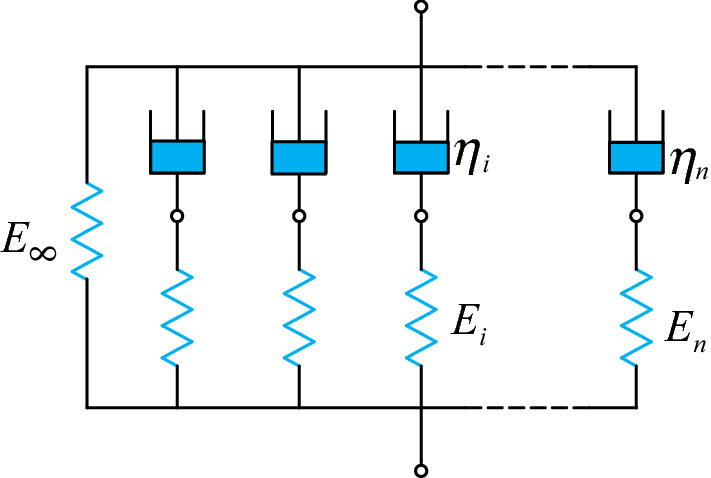
Schematic diagram of generalized Maxwell model.

For the one-dimensional condition, the linear viscoelastic model with relaxation modulus described by the Prony series, namely the generalized Maxwell model (see Fig. 7), can be presented as:

29 $$\tilde{\sigma } = \sigma_{{{\text{linear}}}} { = }\int_{ - \infty }^{t} {E(t - \tau )} \frac{\partial \varepsilon }{{\partial \tau }}{\text{d}}\tau$$

30 $$E(t) = E_{\infty } + \sum\limits_{i = 1}^{n} {E_{i} \exp \left( { - \frac{t}{{\tau_{i} }}} \right)}$$

where $E_{\infty }$ denotes the long term equilibrium relaxation modulus, $E_{i}$ and $\tau_{i}$ mean the *i*_th_ term relaxation modulus and the corresponding characteristic time, respectively.

**Figure 8:**
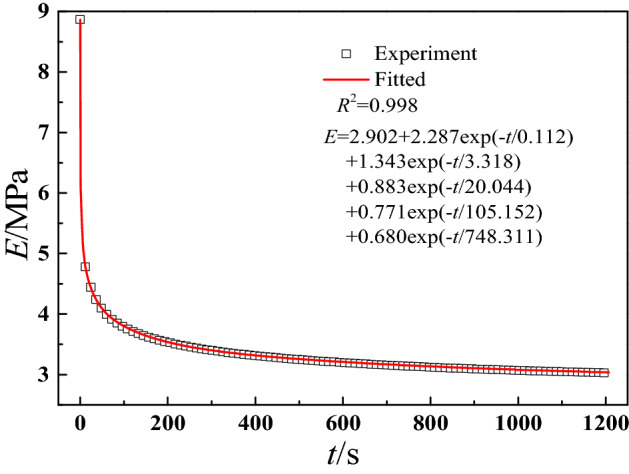
The relaxation modulus curve and fitted result.

It can be found that the linear viscoelastic parameters ($E_{\infty }$, $E_{i}$ and $\tau_{i}$) are crucial to the accuracy of constitutive model in this paper. For ideal results, the stress relaxation tests are requested for a step-strain. However, it is impossible to meet the request using general experimental system, resulting in smaller than actual result. Therefore, a few researchers proposed various methods to obtain a better relaxation modulus result. In this paper, the fitting method based on Prony series proposed by Xu et al.^49^ was used to acquire parameters $E_{\infty }$,$E_{i}$ and $\tau_{i}$. In general, the number of $E_{i}$ and $\tau_{i}$ increase the accuracy of the fitting model, and up to 20 terms Prony series are used for polyimide HFPE-II-52^50^. However, larger terms would lead ill-conditioned identification problems and are not easy to be applied due to complex parameters. For solid propellant, there are usually 3 to 9 terms of Prony series are used to fit relaxation curve at room temperature or master relaxation curve under unsteady temperatures^5,24,32,44,49^. Besides, our recent study has shown that the relaxation curve at a signal temperature has a better prediction accuracy than the master curve under unsteady temperatures^51^. Therefore, in this work, the temperature is uncoupled. To avoid complex parameters, the 5 terms of Prony series are used to fit relaxation curve at room temperature, as shown in Fig. 8. Figure 8 shows a good fitted result.

#### Identification of damage initiation parameter $S_{0}$

The damage initiation parameter $S_{0}$ is defined as the critical point for the transition from a linear response to a nonlinear response. In reality, the method for acquiring the damage initiation parameter is same as the method for obtaining the linear viscoelastic limit stress. One of the common methods is to compare the experimental characteristic curve and ideal linear characteristic stress–strain curve by using the double logarithmic axes^52^. For uniaxial tensile constant-strain-rate test, $\frac{\partial \varepsilon }{{\partial \tau }}{ = }\dot{\varepsilon }$, the ideal linear characteristic curve can be presented as

31 $$\frac{{\sigma_{{{\text{linear}}}} }}{{\dot{\varepsilon }}} = E_{\infty } t + \sum\limits_{i = 1}^{n} {E_{i} \tau_{i} \left[ {1 - \exp \left( { - \frac{t}{{\tau_{i} }}} \right)} \right]}$$

For the one-dimensional condition, the Eq. (18) can be reduced to

32 $$\begin{gathered} Y = \int_{ - \infty }^{\varepsilon } {\sigma_{{{\text{linear}}}} {\text{d}}\varepsilon } \\ { = }\frac{1}{2}E_{\infty } \varepsilon^{2} + \sum\limits_{i = 1}^{n} {\left[ {E_{i} \dot{\varepsilon }\tau_{i} \varepsilon + E_{i} \left( {\dot{\varepsilon }\tau_{i} } \right)^{2} \exp \left( { - \frac{\varepsilon }{{\dot{\varepsilon }\tau_{i} }}} \right)} \right]} \\ \end{gathered}$$

**Figure 9:**
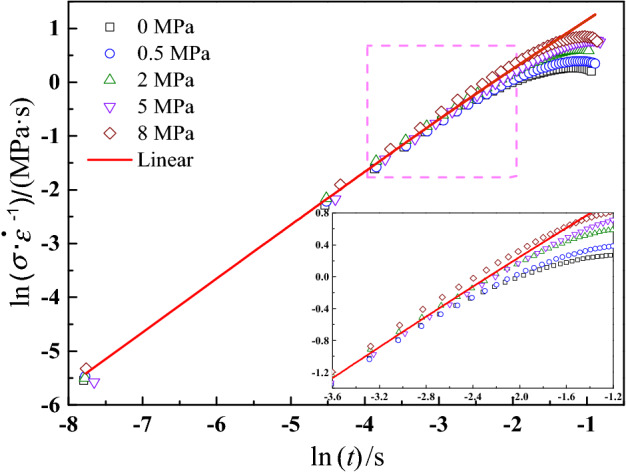
Comparisons of experimental characteristic curves and linear characteristic curve at 1.190 s^−1^.

**Figure 10:**
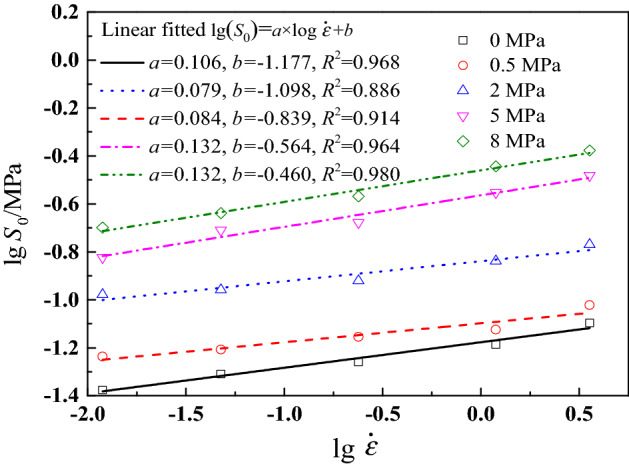
Damage initiation parameter $S_{0}$ under different strain rates and confining pressures.

Figure 9 presents the comparisons of experimental characteristic curves and ideal linear characteristic curve under 1.190 s^−1^ and various confining pressures. The scatter points are experimental data, and the red solid curve is linear characteristic curve calculated through the Eq. (31) and linear viscoelastic parameters shown in Fig. 8. As shown in Fig. 9, under the small strain condition, the experimental characteristic curves and ideal linear characteristic curve are overlapped well, which means the mechanical responses of this stage for CSP can be described by linear viscoelastic theory. As the strain increases, damage begins to accumulate, the nonlinear mechanical behaviors become more prominent, and the experimental characteristic curves gradually deviate from the linear characteristic curve. Also, it can be found that the experimental characteristic curve under 0 MPa is firstly separated from linear characteristic curve, and finally experimental characteristic curve under 8 MPa is separated from linear characteristic curve, which indicates damage initiation points under different confining pressures are different. By analyzing these deviation points and using Eq. (32), damage initiation parameter $S_{0}$ at various experimental conditions can be obtained and presented in Fig. 10.

In fact, the damage initiation parameter $S_{0}$ can be also interpreted as the total input work $W_{{{\text{LVE}}}}$ by the applied stress and strain in the region of linear viscoelastic. Due to the smallness of the dissipated energy compared with the stored one, Brüller^53^ proposed a general energy relation for the case of quasi-elastic linear approximation

33 $$W_{{{\text{LVE}}}} = C\frac{{\sigma_{{{\text{linear}}}} \varepsilon_{{{\text{linear}}}} }}{2}$$

where $\sigma_{{{\text{linear}}}}$ and $\varepsilon_{{{\text{linear}}}}$ are the limit stress and strain of linear viscoelasticity, respectively. The parameter $C$ represents the contribution of time-dependent components to the total energy.

Based on above definition, Starkova et al.^52^ found that $W_{{{\text{LVE}}}}$ cannot be influenced by strain rate and temperature, and it can be considered as a material characteristic. However, it should be noted that the range of strain rate is small in the literature^52^. In this work, Fig. 10 reveals that damage initiation parameter $S_{0}$ is rate-dependent and pressure-dependent. Damage initiation parameter shows a logarithmic relationship with strain rate, whose variation law is similar to the maximum tension strength of solid propellant^46^. Besides, it can be seen that as strain rate and confining pressure increase, damage initiation parameter increases. It also reveals that confining pressure has a delay effect on damage initiation of CSP. The reason is that under confining pressure condition, the particle–matrix interface is tightly compressed by the surrounding pressure, and greater input strain energy is required to achieve particle–matrix interface separation. Under 8 MPa and 3.571 s^−1^, the value of $S_{0}$ is 0.42 MPa, while it is 0.042 MPa under 0 MPa and 1.190 × 10^–2^ s^−1^, which demonstrates that with the coupled effects of strain rate and pressure, the value of $S_{0}$ at 8 MPa and 3.571 s^−1^ is 10 times of its value at 0 MPa and 1.190 × 10^–2^ s^−1^. Therefore, damage initiation parameter can be regarded as a viscoelastic parameter for CSP.

**Figure 11:**
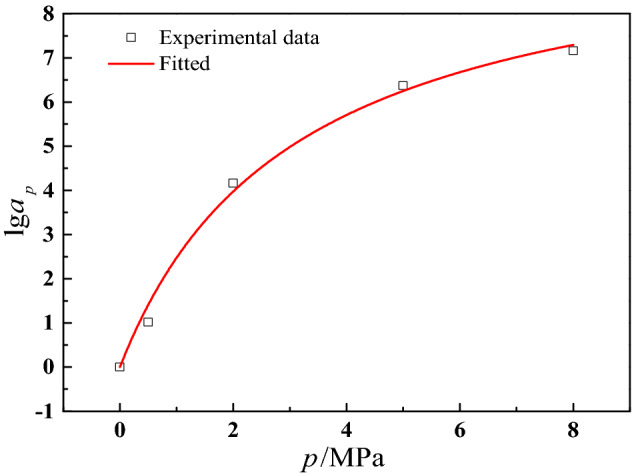
The pressure shift factor fitted result.

Nantasetphong et al.^54^ pointed out that an increase in pressure is related to a decrease in temperature, which means the effect of temperature drop on viscoelastic materials is approximately equal to the increase of confining pressure. The TTSP is widely used to construct the master curves of viscoelastic mechanical parameters of various viscoelastic materials. Meantime, as we discussed above, the variation law of damage initiation parameter with strain rate and confining pressure is similar to maximum tension strength. To describe and predict damage initiation parameter under different strain rates and confining pressures, a master curve of damage initiation parameter should be constructed. Thence, with reference to the application of TTSP to solid propellant^55^, we adopt time-pressure superposition principle^56^ (TPSP) to construct a master curve of damage initiation parameter. The room pressure (0 MPa) sets as reference pressure, and other test curves under various confining pressures are translated along the logarithmic strain rate axis until they overlap with the curve representing the mechanical behavior of propellant under the reference pressure level. The translation distance is defined as the pressure shift factor ${\text{l}}g\alpha_{p}$, which can be expressed as^56^

34 $$\lg \alpha_{p} { = }\frac{{C_{3} (p - p_{{{\text{ref}}}} )}}{{C_{4} - (p - p_{{{\text{ref}}}} )}}$$

where $p$ and $p_{{{\text{ref}}}}$ denote experimental confining pressure and reference confining pressure, respectively. $C_{3}$ and $C_{4}$ are material parameters, which can be obtained by fitting experimental data. The pressure shift factor ${\text{l}}g\alpha_{p}$ is shown in Fig. 11 and the fitted material parameters are listed in Table 1.

**Table 1:** The fitted parameters of shift factor.

Parameters	$C_{3}$	$C_{4}$	$R^{2}$
Values	− 10.104	− 3.083	0.993

**Figure 12:**
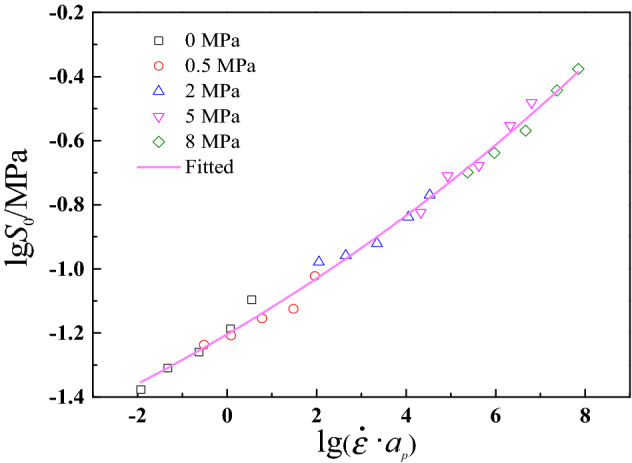
Master curve of damage initiation parameter $S_{0}$ for CSP.

The translation result is shown in Fig. 12, and the relationship between damage initiation parameter and reduced strain rate $\lg (\dot{\varepsilon } \cdot \alpha_{p} )$ can be described by the following formula^57^,

35 $$\lg S_{0} = A_{1} + \frac{{A_{2} }}{{1 + \exp (A_{3} \cdot \lg (\dot{\varepsilon } \cdot \alpha_{p} ) + A_{4} )}}$$

where $A_{1}$, $A_{2}$, $A_{3}$ and $A_{4}$ are best-fitted parameters, which are presented in Table 2. As shown in Fig. 12, the mater curve can describe damage initiation parameter $S_{0}$ under various experimental conditions well, which can be used to predict damage initiation condition of CSP under other strain rates and confining pressures.

**Table 2:** The best-fitted parameters of mater curve of $S_{0}$ for CSP.

Parameters	$A_{1}$	$A_{2}$	$A_{3}$	$A_{4}$	$R^{2}$
Values	45.732	− 48.266	0.0634	− 3.564	0.991

#### Identification of damage model parameters

**Figure 13:**
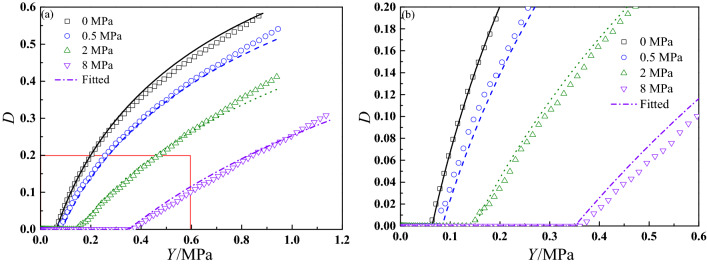
Comparisons of experimental damage evolution curves and fitted curves by Eq. (38) at 1.190 s^−1^ and various confining pressures.

According to the Eq. (13), the isotropic scalar damage variable $D$ can be evaluated using the Eq. (36)

36 $$D = 1 - \frac{\sigma }{{\tilde{\sigma }}} = 1 - \frac{{\sigma_{\text{experiment}} }}{{\sigma_{{{\text{linear}}}} }}$$

where $\sigma_{\text{experiment}}$ and $\sigma_{{{\text{linear}}}}$ are experimental stress result and linear viscoelastic stress, respectively. The damage evolution curves can be obtained through the Eq. (36) at 1.190 s^−1^ and different confining pressures (0 MPa, 0.5 MPa, 2 MPa, 8 MPa), as shown in Fig. 13.

Assuming that the data acquisition frequency is high enough and the time increment $\Delta t$ is small, the derivative of damage variable $D$ to time $t$ (see Eq. (28)) can be expressed in an incremental form as

37 $$\frac{\Delta D}{{\Delta t}} \approx \left[ {\frac{{k_{2} }}{{k_{1} \cdot }} \cdot \left( {\frac{Y(t + \Delta t)}{{S_{0} }}} \right)^{{k_{1} }} \cdot \left( {\frac{{\dot{\varepsilon }}}{{\dot{\varepsilon }_{0} }}} \right) \cdot (1 - D(t))^{n} \cdot \left[ {1 - w \cdot \left( {1 - \exp \left( { - \frac{p}{{p_{0} }}} \right)} \right)} \right]} \right]$$

Furthermore, the value of damage variable at the time of $t + \Delta t$ can be expressed as

38 $$D(t + \Delta t) = \left[ {\frac{{k_{2} }}{{k_{1} \cdot }} \cdot \left( {\frac{Y(t + \Delta t)}{{S_{0} }}} \right)^{{k_{1} }} \cdot \left( {\frac{{\dot{\varepsilon }}}{{\dot{\varepsilon }_{0} }}} \right) \cdot (1 - D(t))^{n} \cdot \left[ {1 - w \cdot \left( {1 - \exp \left( { - \frac{p}{{p_{0} }}} \right)} \right)} \right]} \right] \cdot \Delta t + D(t)$$

where $\dot{\varepsilon }_{0}$ = 1 s^−1^ and $p_{0}$ = 1 MPa. Using the Eq. (38) and experimental damage evolution curves shown in Fig. 13, the optimization objective function as the Eq. (39) is established.

39 $$\left\{ \begin{gathered} \min {\kern 1pt} {\kern 1pt} {\kern 1pt} {\kern 1pt} {\kern 1pt} {\kern 1pt} {\kern 1pt} {\kern 1pt} {\kern 1pt} {\kern 1pt} {\kern 1pt} {\kern 1pt} {\kern 1pt} {\kern 1pt} {\kern 1pt} {\kern 1pt} {\kern 1pt} F(k_{1} ,k_{2} ,n,w) = \sum\limits_{i = 1}^{K} {\sum\limits_{j = 1}^{L} {(D_{{\text{e}}}^{ij} - D_{{\text{t}}}^{ij} )^{2} } } \hfill \\ s.t.{\kern 1pt} {\kern 1pt} {\kern 1pt} {\kern 1pt} {\kern 1pt} {\kern 1pt} {\kern 1pt} {\kern 1pt} {\kern 1pt} {\kern 1pt} {\kern 1pt} {\kern 1pt} {\kern 1pt} {\kern 1pt} {\kern 1pt} {\kern 1pt} {\kern 1pt} {\kern 1pt} {\kern 1pt} {\kern 1pt} {\kern 1pt} k_{1} ,k_{2} ,n,w > 0{\kern 1pt} \hfill \\ \end{gathered} \right.$$

where $D_{{\text{e}}}^{ij}$ is experimental damage variable, and $D_{{\text{t}}}^{ij}$ is numerical solution calculated by Eq. (38). $K$ is the number of the confining pressure levels, $K{ = }4$, i.e., 0 MPa, 0.5 MPa, 2 MPa and 8 MPa, and $j$ is the number of data points at a certain confining pressure condition. The global optimization genetic algorithm in MATLAB R2018a is used to optimize the objective function, and the optimized damage parameters are listed in Table 3. During the damage model parameters optimization process, damage initiation parameter should be calculated by Eq. (34) and (35).

**Table 3:** Damage model parameters of CSP.

Parameters	$k_{1}$	$k_{2}$	$n$	$w$
Values	0.153	0.317	1.238	0.382

Figure 13 shows the comparisons of experimental damage evolution curves and fitted results, which indicate the energy-based damage evolution model developed in this work can describe the experimental results well. From the figure, the damage growth rate decreases with increasing confining pressure. Under a same strain energy density, the value of damage gradually decreases as confining pressure increases. For example, when the strain energy density is 0.5 MPa, the value of damage is 0.41 at 0 MPa, while it is 0.07 at 8 MPa. In conclusion, confining pressure shows a significant suppression effect on the damage growth and the proposed damage evolution model can describe the damage growth behaviors of CSP under different confining pressures.

### Model validation

**Figure 14:**
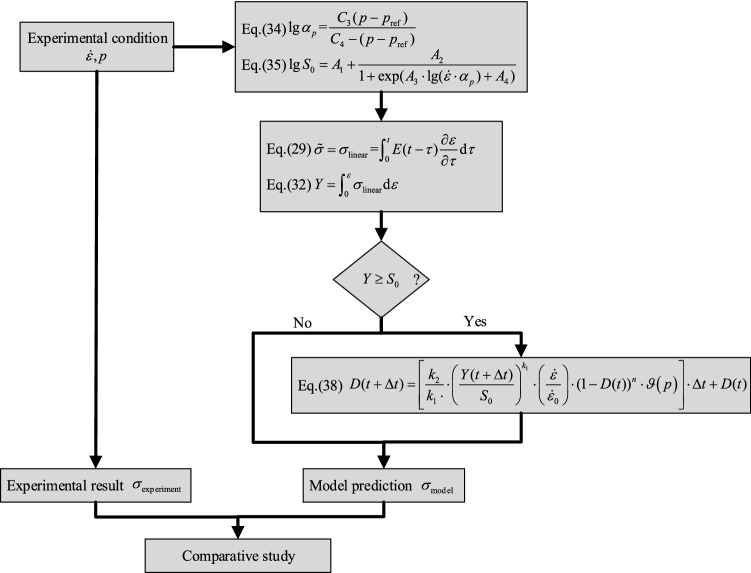
The validation process of constitutive model.

In this section, uniaxial constant rate tensile tests and uniaxial dual rates tensile tests will be used to validate the accuracy of the constitutive model. The model validation process are divided into four steps, including damage initiation parameter $S_{0}$ calculation, linear viscoelastic stress $\sigma_{{{\text{linear}}}}$ and linear viscoelastic strain energy density $Y$ calculation, damage variable calculation $D$ and stress $\sigma_{\text{model}}$ calculation. The flow chart of validation process is shown in Fig. 14 and performed through MATLAB R2018a.

#### Uniaxial constant rate tensile tests

**Figure 15:**
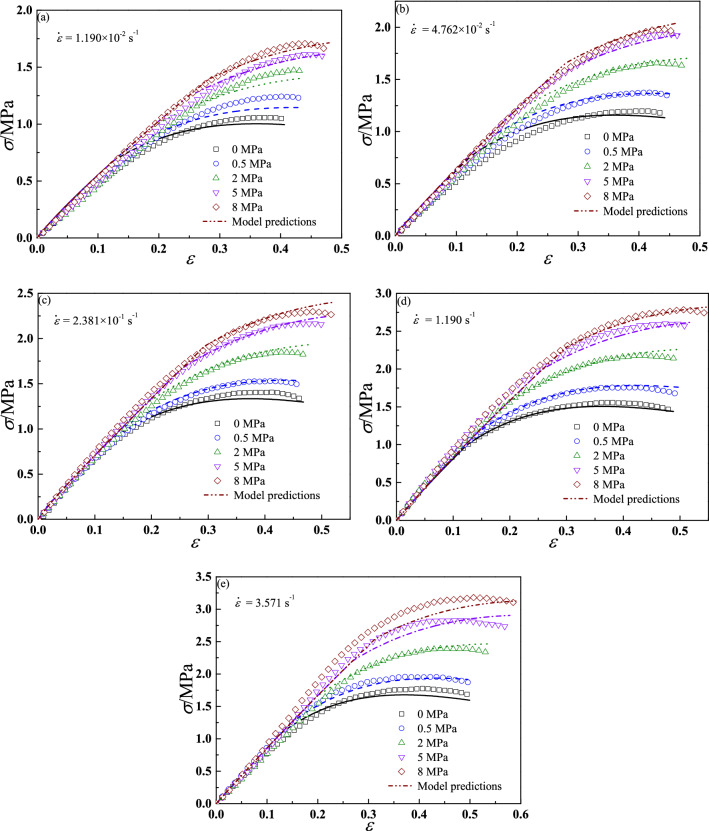
Comparisons between experimental results and model predictions.

Figure 15 shows comparisons between experimental results and model predictions under different experimental conditions. It should be noted that the experimental results at 1.190 s^−1^ are used to identify the damage model parameters in section “Identification of damage model parameters”, and other prediction results are calculated for the identified parameters. The figure shows a good overlap between experimental results and model predictions.

**Figure 16:**
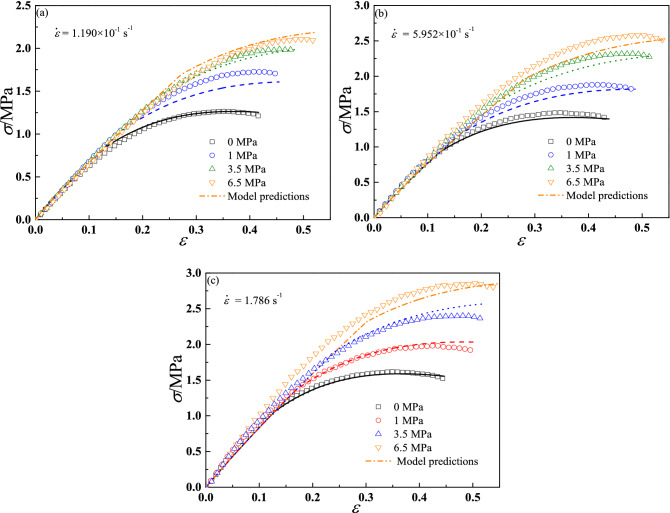
The validation results of tensile constant strain rate tests.

However, in seriously speaking, the above validation results (Fig. 15) can only prove the accuracy of damage model parameters, the accuracy of master curve of damage initiation parameter $S_{0}$ cannot be proved due to the above experimental data were used for identification of damage initiation parameter in section “Identification of damage initiation parameter”. Therefore, another three groups of tension tests of 500 mm/mm、2500 mm/min and 7500 mm/min (the corresponding strain rates is 1.190 × 10^–1^ s^−1^、5.952 × 10^–1^ s^−1^ and 1.786 s^−1^) at relative atmospheric pressure of 0 MPa (room pressure), 1 ± 0.05 MPa, 3.5 ± 0.05 MPa and 6.5 ± 0.1 MPa were also performed to validate the accuracy of the proposed constitutive model. The experimental temperature is same with the above tests. Figure 16 presents the prediction results have a good agreement with experimental data.

#### Uniaxial dual rates tensile tests

**Figure 17:**
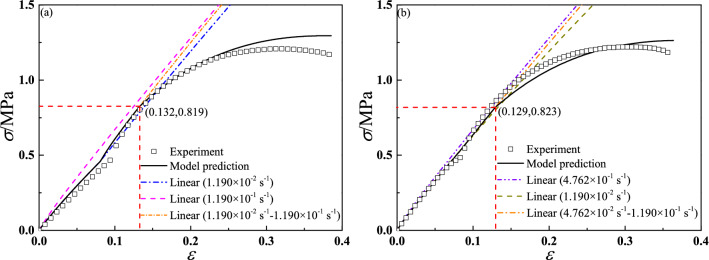
The validation results of tensile dual strain rates tests.

Due to dual strain rates procedure was not designed in new experimental system, the dual strain rates tests were carried out through electronic universal testing machine (QJ211)^1^ at room pressure. The first group of test is that the CSP specimen was initially loaded at 1.190 × 10^–2^ s^−1^, upon reaching a strain of 0.08, and the strain rate increased to 1.190 × 10^–1^ s^−1^. The second group of test is that the CSP specimen was initially loaded at 4.762 × 10^–2^ s^−1^, upon reaching a strain of 0.08, and the strain rate increased to 1.190 × 10^–1^ s^−1^. The experimental temperature is same with the above tests. Figure 17 shows the validation results of dual strain rates tests. It can been seen that the model predictions agree with experimental results well. It proves the constitutive model has a good predictive capability under a wide experimental condition.

### Model evaluation

In order to assess the predictive capacity of proposed model, the root mean square errors (RMSE) is introduced and calculated as follows

40 $$RMSE = \sqrt {\frac{1}{q}\sum\limits_{i = 1}^{q} {\left( {\sigma_{\text{experiment}} - \sigma_{\text{model}} } \right)^{2} } }$$

where $\sigma_{\text{experiment}}$ denotes the experimental maximum tensile strength of CSP, $\sigma_{\text{model}}$ is the model prediction corresponding to the same strain, and $q$ is number of data points, $q = 1000$.

Table 4 shows the values of RMSE under different confining pressures and strain rates. It can be seen that the maximum value of RMSE is 0.157 MPa and the most cases are lower than 0.1 MPa, which demonstrate the constitutive model has a good ability to describe the coupled effects of confining pressure and strain rate on nonlinear stress-stain relationships of CSP.

**Table 4:** The values of RMSE under various experimental conditions.

Strain rate/s^−1^	0 MPa	0.5 MPa	2 MPa	5 MPa	8 MPa
1.190 × 10^–2^	0.0565	0.0674	0.0719	0.0513	0.0270
4.762 × 10^–2^	0.0714	0.0597	0.0627	0.0342	0.0319
2.381 × 10^–1^	0.0476	0.0343	0.0441	0.0249	0.0469
1.190	0.0319	0.0323	0.0460	0.0785	0.0728
3.571	0.0731	0.0344	0.0700	0.0867	0.157

Strain rate/s^−1^	0 MPa	1 MPa	3.5 MPa	6.5 MPa	
1.190 × 10^–1^	0.0287	0.0903	0.0293	0.0503	
5.952 × 10^–1^	0.0459	0.0760	0.0840	0.123	
1.786	0.0358	0.0561	0.0837	0.148	

## Discussions

By developing a confining pressure experimental system, we obtained the mechanical properties of CSP. The mechanical properties are same with previous research results, that is, with increasing confining pressure, the maximum tensile stress increases^11,22,46–48^. Nevertheless, there is a new phenomenon is observed that at low strain rate loading (see Fig. 15a and b, and Fig. 16a), these is a small difference in stress results between 5 and 8 MPa due to the existence of saturation confining pressure, while at medium strain rate loading (see Fig. 15e and Fig. 16c), this difference increases obviously. It demonstrates that the value of saturation confining pressure depends on strain rate and is not a constant as the references^46–48^ reported, which deserves a further study. This experimental phenomenon confirms Traissac’s^45^ conclusion that the saturation confining pressure depends on experimental conditions. In addition, the experimental results shows the stress–strain curves of CSP under a wide range strain rates (1.190 × 10^–2^ s^−1^–3.571 s^−1^) and confining pressure conditions (relative atmospheric pressure of 0 MPa-8 MPa), and it is expected that these results will provide experimental verification support for other researchers’ research on constitutive models for CSP.

By proposing an energy-based damage evolution model considering the coupled effects of strain rate and confining pressure and incorporating it into linear viscoelastic model, we successfully describe the stress–strain properties of CSP at different strain rates and confining pressures, as shown in Figs. 15 and 16. It can be found that comparing to previous results^11,12,20,21,24^, the model parameters identification processes in our model are simpler and easier to be conducted by tensile tests. From the Fig. 17, it proves the good predictive ability of energy-based damage initiation criterion comparing to stress-based or strain-based damage initiation criterion. If stress-based or strain-based criteria was used in this work, the damage initiation point would be consistent under both dual strain rates loading conditions due to they do not consider the loading history. Obviously, the two different loading conditions should have different damage initiation points, and energy-based damage initiation criterion can predict it well, especially for the wide range of strain rates.

However, there are some poor predictions (see Table 4) can be observed, which may be caused by following three reasons. Firstly, the linear viscoelastic parameters obtained thorough a simple stress relaxation test seem to be not ideal results, which are difficult to describe the linear viscoelastic behaviors under a wide range of strain rates (see Fig. 15a). However, in our model, the damage driving force and linear stage of stress–strain curve are calculated by linear viscoelastic parameters, which may lead a large error. It can be inferred that the better linear viscoelastic parameters will increase the accuracy of model predictions, e.g. Park’s^11^ result. Secondly, the damage initiation parameter $S_{0}$ also plays an important role in predicting the transition point from linear to nonlinear response and damage evolution in this work. Although the master curve can describe the damage initiation parameter under different experimental conditions, there is still a certain error, resulting in the final poor prediction of stress–strain curves (see Figs. 15e and 16b). If the damage initiation parameter less than the ideal result, which would lead to a larger damage variable and a smaller stress result, otherwise a smaller damage variable and a larger stress result would be obtained. Thirdly, as we discuss above that the value of saturated confining pressure seems to be related to strain rate, we do not consider this experimental phenomenon in the proposed model, which may cause the prediction errors at high strain rate and confining pressure, and it also needs a further study.

Obviously, environmental temperature also affects the mechanical performance of solid propellant much. With the coupled effects of strain rate, confining pressure and temperature, its stress–strain relation will become more complex. However, the constitutive model study coupled effects of these three factors will provide a strong support for the structural integrity analysis of SRM. As did by Chen et al.^37^ and Abu Al-Rub et al.^38^, the Arrhenius-type equation can be added to our model to describe the effect of temperature.

## Conclusions

In this work, based on thermodynamic theory and continuous damage mechanics theory, a nonlinear viscoelastic model of CSP considering strain rate and confining pressure was proposed and corresponding model parameters identification process were presented. The key idea of the model was to develop a viscodamage model by introducing linear viscoelastic strain energy density as the damage driving force, and taking the coupled effects of strain rate, damage history and confining pressure on damage growth into account. Comparing to experimental results from low to medium strain rates, and low to high confining pressures, the model predictive capability was demonstrated. Conclusions of this study can be summarized as follows:

1. The mechanical properties of CSP are significantly influenced by the strain rate and confining pressure. As confining pressure and strain rate increase, the maximum tensile strength increases. The value of saturation confining pressure is related to strain rate.
2. Confining pressure has a significant suppression effect on the damage initiation and evolution. With the increase of strain rate and confining pressure, the damage initiation parameter increases. The energy-based damage initiation parameter can be considered as a viscoelastic parameter for CSP. Based on the time-pressure superposition principle, the master curve of damage initiation parameter was constructed and can present damage initiation condition of CSP under various experimental conditions well.
3. By comparing model predictions with uniaxial constant rate tests and dual rates tests, the maximum value of RMSE is 0.157 MPa and most cases are lower than 0.1 MPa, which proves the nonlinear viscoelastic model with damage shows a good predictive capability.

## Data Availability

The datasets used during the current study available from the corresponding author on reasonable request.
